# Role of TRPM7 in Cancer: Potential as Molecular Biomarker and Therapeutic Target

**DOI:** 10.3390/ph10020039

**Published:** 2017-04-05

**Authors:** Nelson S. Yee

**Affiliations:** Division of Hematology-Oncology, Department of Medicine, PennState Health Milton S. Hershey Medical Center, Program of Experimental Therapeutics, PennState Cancer Institute, The Pennsylvania State University College of Medicine, 500 University Drive, Hershey, PA 17033, USA; nyee@pennstatehealth.psu.edu; Tel.: +1-717-531-0003

**Keywords:** transient receptor potential, TRP, TRPM7, ion channel, cancer, biomarker, therapeutic target

## Abstract

The transient receptor potential melastatin-subfamily member 7 (TRPM7) is a ubiquitously expressed ion channel with intrinsic kinase activity. Molecular and electrophysiological analyses of the structure and activity of TRPM7 have revealed functional coupling of its channel and kinase activity. Studies have indicated the important roles of TRPM7 channel-kinase in fundamental cellular processes, physiological responses, and embryonic development. Accumulating evidence has shown that TRPM7 is aberrantly expressed and/or activated in human diseases including cancer. TRPM7 plays a variety of functional roles in cancer cells including survival, cell cycle progression, proliferation, growth, migration, invasion, and epithelial-mesenchymal transition (EMT). Data from a study using mouse xenograft of human cancer show that TRPM7 is required for tumor growth and metastasis. The aberrant expression of TRPM7 and its genetic mutations/polymorphisms have been identified in various types of carcinoma. Chemical modulators of TRPM7 channel produced inhibition of proliferation, growth, migration, invasion, invadosome formation, and markers of EMT in cancer cells. Taken together, these studies suggest the potential value of exploiting TRPM7 channel-kinase as a molecular biomarker and therapeutic target in human malignancies.

## 1. Introduction

Ion channels play oncogenic and tumor suppressive roles in the pathogenesis of malignant neoplasms, and they have been implicated in the various hallmarks of cancer [1]. The transient receptor potential (TRP) superfamily of protein function as channels that control passage of various ions across biological membranes [2]. Activation of TRP channels typically results in transmembrane flow of cations such as Ca^2+^ and Mg^2+^ and, consequently, modulation of the associated signaling pathways. By detecting changes in various physical and chemical stimuli, TRP channels act as cellular sensors and transducers and mediate a variety of physiological responses [3]. Of the eight sub-families in the vertebral TRP family, the melastatin-subfamily (TRPM) possesses unique structural motifs and share common architectural features [4]. Growing evidence has shown that the TRPM7 member plays crucial roles in cellular processes, embryonic development, and human diseases, particularly cancer [5,6,7,8]. Accumulating data suggest the potential value of TRPM7 as a molecular biomarker and therapeutic target in human malignancies [5,8,9].

**Figure 1:**
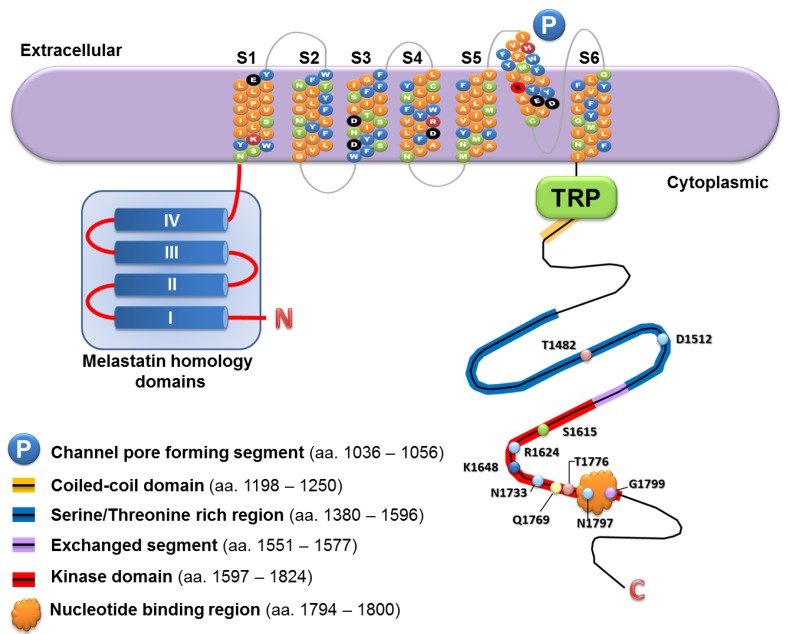
A schematic diagram to illustrate the protein structure of TRPM7 channel-kinase.

The biochemical and electrophysiological properties of TRPM7 have been determined by in vitro assays, and the functional roles of TRPM7 have been studied in cultured cells and model organisms. Under physiological conditions, TRPM7 is a divalent cation-selective channel, and it possesses protein serine/threonine kinase activity [10,11,12]. The TRPM7 channel-kinase is ubiquitously expressed [13]. Spanning over 134.34 kb on the long arm of chromosome 15, the human *TRPM7* gene consists of 39 exons, and four transcripts of its nine splice variants encode protein. The full-length human TRPM7 transcript contains 7263 nucleotides, and the encoded protein is composed of 1865 amino acids (MW 210 kDa) [14]. The basic structural features of the TRPM7 protein are shown in Figure 1. As a regulator of ionic homeostasis, the TRPM7 channel preferentially permits the flow of Mg^2+^ and Ca^2+^, and the Mg^2+^ influx through the TRPM7 channel in certain cell types can lead to altered intracellular levels of Ca^2+^ [12,15,16]. The physiologically essential divalent metal cations ( Zn^2+^, Mn^2+^, Co^2+^) as well as environmentally toxic metals (Ni^2+^, Cd^2+^, Ba^2+^, Sr^2+^) are also permeable through the TRPM7 channel [10,12,17]. In addition, as a member of the atypical protein kinase family called alpha-kinases [18], TRPM7 can autophosphorylate its serine and threonine residues [19]. The roles of the channel and kinase activities in the physiological functions of TRPM7 depend on the cell types and the molecular context.

The TRPM7 protein contains six transmembrane segments (S1 to S6), each about 21 amino acid (aa.) residues in length. The amino (N) and carboxyl (C) terminal components embrace the transmembrane segments. The channel pore (P) is shown slightly off plane and formed between S5 and S6. The two negatively charged amino acids (E1047 and E1052) in the pore forming loop are important for Ca^2+^ and Mg^2+^ permeability as well as pH sensitivity of the channel. Selected amino acid residues are shown and they play important roles in the functions of TRPM7 channel and kinase. The numbers of the amino acid residues correspond to human TRPM7 protein. This figure is adapted from *Cells* 2014, *3*, 751–777 with permission from the publisher [5].

Experimental analyses of TRPM7 channel-kinase have revealed the molecular features that are important for its physiological responses and biological functions of TRPM7 in normal and cancerous cells. In vitro studies using site-directed mutagenesis in combination with electrophysiological and biochemical studies have generated insights into the molecular basis underlying the TRPM7 channel-kinase activities. Moreover, the amino acid residues of the TRPM7 protein that modulates its kinase activity, the sites of autophosphorylation and substrate binding, and their functional significance have been determined [5,20]. A growing body of data have supported diverse roles of TRPM7 in cellular proliferation, survival, differentiation, growth, and migration [5,21,22]. Studies using model organisms have revealed the crucial roles of TRPM7 in embryogenesis as well as the requirement of TRPM7 for development and functions of melanocytes, skeleton, thymus, nervous system, kidney, exocrine pancreas, urinary bladder, and megakaryocytes/platelets [5,23,24,25,26]. Accumulating evidence indicates that the TRPM7 channel-kinase plays oncogenic and tumor suppressing roles in various types of malignant tumors.

This purpose of this article is to discuss the emerging roles of the TRPM7 channel-kinase in various human malignancies, and the potential of exploiting TRPM7 as a cancer biomarker and therapeutic target. In this article, I will provide a review of the expression of TRPM7 in cancer, the roles of TRPM7 in cancer cells including proliferation, survival, migration, invasion, and epithelial-mesenchymal transition, as well as the role of TRPM7 in tumor growth and metastasis. The findings of TRPM7 genetic polymorphisms and mutations in various carcinoma will be presented. A list of chemicals that modulate TRPM7 expression and/or channel activity will be summarized. Finally, I will discuss the potential of developing TRPM7 channel-kinase as a molecular biomarker and therapeutic target for achieving the goal of precision oncology.

## 2. Roles of TRPM7 in Human Cancer

The TRPM7 channel-kinase has been implicated in a variety of human malignant tumors. In certain carcinoma examined, TRPM7 is aberrantly over-expressed in cell lines and/or tissues. Consistent with the functional roles of TRPM7 in the normal cell types and during organogenesis, numerous studies have shown that TRPM7 regulates cellular proliferation, survival, cell cycle progression, migration, and invasion in cancer cell lines. The signaling mechanisms underlying the biological functions of TRPM7 have been elucidated, and how aberrant expression and activity of TRPM7 contributes to neoplasia has begun to be understood. The expression and functional roles of TRPM7 in human malignant diseases are summarized in Table 1.

The functional roles of TRPM7 were examined by using RNA interference-mediated inhibition of TRPM7 expression or by using chemical inhibitors of TRPM7 channel activities as indicated. RNA interference is generally target-specific, though the extent of inhibition and the stability of the interfering agents are issues of potential concern. While the chemical inhibitors are relatively specific for TRPM7 activity, they may produce “off-target” effects depending on their concentrations being used. This table is adapted and modified from *Cells* 2014, *3*, 751–777 with permission from the publisher [5].

### 2.1. Expression of TRPM7 in Cancer

While it is ubiquitously expressed in normal tissues and cells, TRPM7 is aberrantly over-expressed in various types of malignant neoplasms (Table 1). Studies have demonstrated increased expression of TRPM7 in a panel of human pancreatic adenocarcinoma cells and tissues [9,27,28,29,30,31]. For each histological type of pancreatic tumors, the proportions of samples with corresponding TRPM7 expression levels have been reported [31]. The expression levels of TRPM7 in pancreatic adenocarcinoma tissues were found to positively correlate with the primary tumor size and tumor stages. These results suggest that aberrant over-expression of TRPM7 is associated with pancreatic tumor growth and metastasis. Besides pancreatic cancer, TRPM7 is aberrantly over-expressed in the cell lines and tissues of breast cancer [33] and glioblastoma [57]. Somatic mutations or polymorphisms of TRPM7 have been identified in breast carcinoma [40], gastric carcinoma [40], colon carcinoma [52], and ovarian carcinoma [40]. While the significance of those mutations and polymorphisms of TRPM7 remains to be determined, the Thr1482Ile polymorphism was shown to be associated with elevated dietary Ca^2+^:Mg^2+^ ratio and risk of colonic polyps [52].

### 2.2. Roles of TRPM7 in Proliferation of Cancer

The proliferative role of TRPM7 has been demonstrated in a variety of malignant tumors including pancreatic adenocarcinoma, breast carcinoma, head/neck carcinoma, retinoblastoma, and glioblastoma. In studies using human pancreatic adenocarcinoma cell lines, TRPM7 channels have been shown to be necessary for maintaining proliferation and preventing replicative senescence [9,27,30]. Additionally, downregulation of TRPM7 in human pancreatic cancer cells led to inhibition of proliferation by arresting the cells in the G_0_/G_1_ and G_2_/M phases of the cell cycle; these effects could be reversed by Mg^2+^ supplementation [9,27,30,31]. Moreover, small interfering RNA mediated silencing of TRPM7 induced senescence-associated β-galactosidase in pancreatic adenocarcinoma cells, suggesting a novel role of ion channels in replicative senescence of cancer [30]. Besides pancreatic cancer, TRPM7 is required for proliferation of cancer cells derived from a variety of malignant tumors. These include breast carcinoma [33], head/neck carcinoma [45], retinoblastoma [47], prostate carcinoma [56], T cell leukemia and rat basophilic leukemia [53], hypopharyngeal squamous cell carcinoma [46], and glioblastoma [57,58,59]. These results indicate that TRPM7 is required for proliferation of cancer cells and support a potential role of TRPM7 channels in tumor growth.

### 2.3. Roles of TRPM7 in Survival of Cancer Cells

A pro-survival role of TRPM7 channels has been demonstrated in various cancer cells, including pancreatic adenocarcinoma, gastric carcinoma, and breast carcinoma. In pancreatic cancer cells, small interfering RNA (siRNA)-induced knockdown of TRPM7 induced cell death without causing apoptosis [30]. In gastric cancer cells, TRPM7 is required for Mg^2+^-dependent cell survival, and involved in ginsenoside Rd-induced apoptosis [41,42,43]. In breast cancer cells, TRPM7 in involved in ginsenoside Rd-induced apoptosis [37]. Results of these studies suggest the mechanisms that mediate TRPM7-induced cell death may depend on the cell types; however, they support a role of TRPM7 in promoting survival of cancer cells and tumor growth.

### 2.4. Roles of TRPM7 in Migration and Invasion of Cancer Cells

Downregulation of TRPM7 in human cancer cells impaired cell migration and invasion; these effects could be reversed by Mg^2+^ supplementation. TRPM7 is required for cell migration in breast carcinoma that involves the kinase domain of TRPM7 as well as phosphorylation of Src and MAPK [32,34,36]. Similarly, TRPM7 is required for cell migration in nasopharyngeal carcinoma [44] and lung carcinoma [50]. In pancreatic adenocarcinoma, TRPM7 is required for Mg^2+^-dependent cell migration [9,28] and cell invasion [31]. These results indicate that TRPM7-regulated Mg^2+^ homeostasis and the associated signaling are required for migration and invasion of cancer cells, and support a potential role of TRPM7 channels in tumor metastasis.

### 2.5. Role of TRPM7 in Epithelial-Mesenchymal Transition

Functional expression of TRPM7 plays a regulatory role in epithelial-mesenchymal transition (EMT), which represents a tumor microenvironment-induced invasive phenotype adopted by cancer cells in metastasis. Epidermal growth factor (EGF)- or hypoxia-induced EMT is associated with a transient elevation of intracellular Ca^2+^ and activation of signal transducer and activator of transcription 3 (STAT3). Silencing of TRPM7 in a breast cancer cell line (MDA-MB468) produced suppression of EGF-induced expression of vimentin and phosphorylation of STAT3, which are markers of EMT [39]. These data suggest that TRPM7 channel is involved in EMT and tumor metastasis.

### 2.6. Roles of TRPM7 in Cancer Growth and Metastasis

In vivo studies have provided insights into the roles of TRPM7 in tumorigenesis. Using a mouse xenograft model of human breast carcinoma, TRPM7 has been shown to be required for tumor metastasis [32]. This process involves TRPM7-mediated modification of focal adhesion number, cell–cell adhesion and polarized cell movement through regulation of myosin II–based cellular tension. These data are consistent with the in vitro evidence for the requirement of TRPM7 in cancer cell migration and invasion and supportive of a mechanosensory role of TRPM7 in tumor metastasis.

### 2.7. Signaling Mechanisms for Functional Roles TRPM7 in Cancer

**Figure 2:**
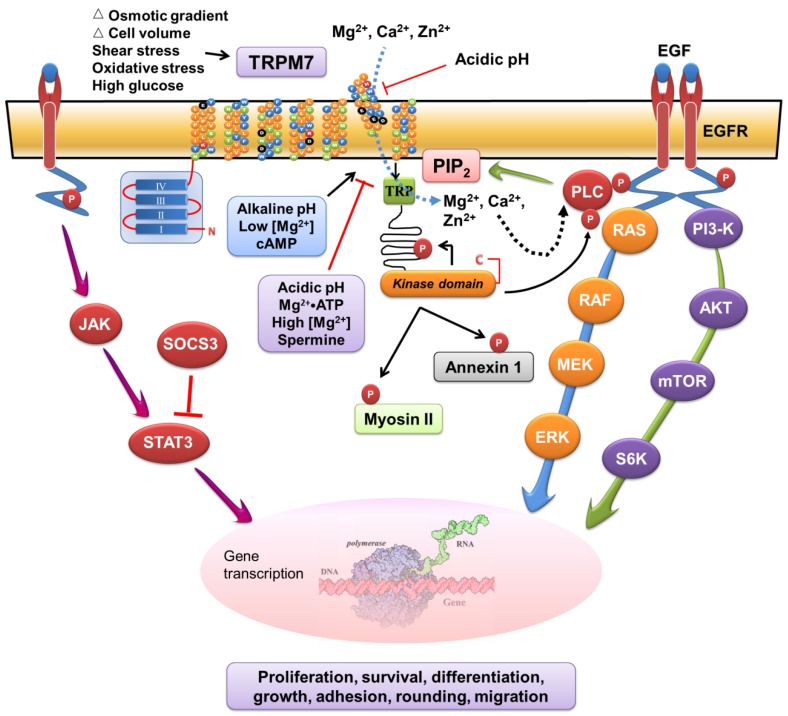
A working model of the signaling mechanisms that mediate the functional roles of TRPM7.

The signaling pathways and the mechanisms that mediate the various cellular effects of TRPM7 in cancer cells have been elucidated. Depending on the cell types, the TRPM7 channel–kinase may interact and modulate the signaling pathways that mediate the effects of mitogens and inflammatory cytokines [22,27,50,58,60,61,62,63,64,65,66,67]. In a working model (Figure 2), the TRPM7 channel-kinase acts as a cellular sensor of the physical and chemical stimuli such as mechanical stretch, oxidative stress, changes in cell volume or osmolar gradient, and alterations in extracellular or cytosolic pH. It also acts as a signal transducer by controlling ionic fluxes and modulating the mitogen- and cytokine-induced signaling pathways. Hypothetically, aberrantly expressed TRPM7 and dysregulated homeostasis of Mg^2+^ and Ca^2+^ in cancer cells modulate the epidermal growth factor (EGF)- or other mitogen-induced signaling pathways. These lead to perturbation of the signaling mediators and nuclear events, resulting in uncontrolled proliferation, survival, growth, and invasion of cancer cells.

The TRPM7 channel and kinase are constitutively active in resting cells. In response to physicochemical stimuli in the extracellular medium and in the cytosol, conduction of Mg^2+^ and Ca^2+^ through the TRPM7 channel can be positively or negatively regulated. The TRPM7 kinase can auto-phosphorylate itself and also phosphorylate various substrates in the cytocol. The resultant perturbation of ionic homeostasis and series of phosphorylation events produce activation or inhibition of the signaling molecules downstream of epidermal growth factor or other cytokines. Those signaling events induce transcription and translation of the cell cycle regulators, senescence-associated genes, and motility factors. These lead to a number of biological processes including cellular survival, proliferation, differentiation, growth, adhesion, rounding, migration, and invasion. Such cellular effects underlie TRPM7 channel-kinase mediated functions in physiological responses, embryonic development, and diseases such as cancer. This figure is adapted and modified from *Cells* 2014, *3*, 751–777 with permission from the publisher [5].

## 3. TRPM7 Channel as Molecular Biomarker and Therapeutic Target in Cancer

The findings of over-expression of TRPM7 protein and genetic mutations/polymorphisms in the TRPM7 gene in (pre)malignant diseases suggest the potential of exploiting it as a cancer biomarker. In pancreatic adenocarcinoma, a positive correlation was identified between the aberrant over-expression of TRPM7 and the tumor size/stages [31]. Results from the epidemiological study demonstrate that the TRPM7 variant T1482I, which was previously identified in patients with neurodegenerative diseases, is associated with dietary intake of Ca^2+^/Mg^2+^ and formation of the pre-malignant colonic adenoma/polyps [52]. Moreover, somatic mutations in TRPM7 have been identified in breast carcinoma (T720S, Thr->Ser), gastric carcinoma (M830V, Met->Val), and ovarian carcinoma (S406C, Ser->Cys) [40]. While the functional significance of these mutations remain to be determined, these data suggest the potential of exploiting TRPM7 as a molecular biomarker for prevention, early detection, and prognostication of cancer.

Furthermore, the aberrant expression and/or activity of TRPM7 in malignant tumors offer the opportunity of targeting TRPM7 for treatment of patients with cancer. A number of chemicals that modulate the expression and channel activity of TRPM7 have been identified and characterized [68]. These are not only valuable research tools to probe the mechanisms underlying the electrophysiological and cellular functions of TRPM7, but also potential therapeutic agents for various diseases, particularly cancer (Table 2).

Those chemical modulators can inhibit the TRPM7 channel activity and/or expression have been studied [37,38,39,42,46,53,54,57,58,59,69,70,71,72,73,74,75,76]. These inhibitors of TRPM7 channels include non-specific channel blockers, compounds derived from natural sources, and synthetic compounds. Most of their inhibitory actions are reversible at the concentrations tested and their IC_50_ values in the µM range. The chemical inhibitors of TRPM7 have been extensively used to study the mechanisms of the TRPM7 channel and kinase, and some of them show potential for therapeutic application.

On the other hand, a set of small molecule chemicals that activate the TRPM7 channel has been identified and characterized [77]. Among these TRPM7 agonists, the δ–opioid receptor antagonist, naltriben, has been studied in detail. It was proposed that naltriben is a positive gating modulator of a TRPM7 channel, with a reversible stimulatory effect on the TRPM7 channel that is independent of [Mg^2+^]_ic_, and an EC_50_ 20.7 µM [77]. In a recent report, two positive modulators of TRPM7, mibefradil and NNC 50-0396, have been recovered from a high throughout screen [78]. Mifebradil was shown to reversibly activate TRPM7-mediated Ca^2+^ entry and whole cell currents. In contrast to naltriben, mifebradil activates the TRPM7 channel only at physiological [Mg^2+^]_ic_. These TRPM7 channel agonists will be useful tools to study the mechanistic actions of TRPM7, and their biological effects and potential medical applications remain to be determined.

Some of the TRPM7 modulators have been tested in cancer cells, such as a clinically used anesthetic (midazolam), naturally occurring compounds (ginsenoside Rg3, ginsenoside Rd, waixenicin A, carvacrol, and xyloketal B), and the synthetic compound NS8593. The chemically induced blockade of TRPM7 expression or its channel activity produces a variety of cellular effects including inhibition of cancer cell survival, proliferation, migration, invasion, and invadosome formation (Table 2). This suggests the potential value of developing these chemical modulators of TRPM7 into anti-cancer therapeutics.

## 4. Conclusions

TRPM7 is a ubiquitously expressed ion channel with intrinsic kinase activity that plays regulatory roles in a variety of cellular processes, physiological responses, early development, organogenesis, and human diseases, particularly cancer. Experimental evidence implicates important roles of the TRPM7 channel-kinase in the hallmarks of cancer, including uncontrolled cell cycle progression, survival, proliferation, growth, migration, invasion, epithelial-mesenchymal transition, and metastasis. While the functions mediated by TRPM7 in cancer appear to depend on the organ involved, the signaling mechanisms that mediate the functional roles of TRPM7 are related to the cellular and molecular context.

Future studies are indicated to understand how the TRPM7 channel-kinase sensing the physical and chemical changes inside the cells and in the microenvironment contributes to neoplasia. Animal models are urgently needed to determine the mechanistic roles of TRPM7 channel-kinase in the multistep process of carcinogenesis, such as tumor initiation, growth, invasion, and metastasis. The aberrant expression of TRPM7 and its genetic mutations/polymorphisms in malignant tumors suggest the opportunity for developing it as a clinical biomarker for prevention and early detection of cancer. Pharmacological inhibition of the TRPM7 channel in conjunction with genetic silencing of TRPM7 expression have not only provided mechanistic understanding of the biological functions of TRPM7, but also offer new hope for developing targeted therapeutics for achieving the goal of precision oncology.

## Figures and Tables

**Table 1:** Expression and roles of TRPM7 channels in various human malignancies.

Cancer	Expression	Functional roles of TRPM7	References
Pancreatic adenocarcinoma	- Increased in human pancreatic adenocarcinoma tissues and cell lines. - Increased in chronic pancreatitis, pancreatic intra-epithelial neoplasms	- Required for cellular proliferation and cell cycle progression involving Mg^2+^. - Required for preventing replicative senescence. - Required for cell migration involving Mg^2+^. - Required for cell invasion.	[9,27,28,29,30,31]
Breast carcinoma	- Over-expression in human breast carcinoma tissues and cell lines - Increased expression in infiltrating ductal carcinoma with microcalcifications - Somatic mutation T720S (Thr→Ser) in a breast infiltrating ductal carcinoma	- Required for cancer cell proliferation in vitro. - Required for cancer cell migration in vitro and tumor metastasis in a mouse xenograft model. Waixenicin A, TRPM7 blocker, inhibits growth and survival of breast cancer cells MCF-7. - TRPM7 involved in estrogen receptor-negative metastatic breast cancer cells migration through kinase domain. - Involved in ginsenoside Rd-induced apoptosis in cells. - Involved in epithelial mesenchymal transition. - TRPM7 mediates migration and invasion of breast cancer cells (MDA-MB-435) involving phosphorylation of Src and MAPK.	[32,33,34,35,36,37,38,39,40]
Gastric carcinoma	- Expressed in human gastric adenocarcinoma cell lines (AGS, MKN-1, MKN-45, SNU-1, SNU-484) - Somatic mutation M830V (Met→Val) in gastric adenocarcinoma	- Required for cell survival involving Mg^2+^. - Waixenicin A, TRPM7 blocker, inhibits growth and survival of gastric cancer cells AGS. - Involved in ginsenoside Rd-induced apoptosis AGS cells.	[37,38,40,41,42,43]
Head and neck Carcinoma	- Expressed in FaDu cells and SCC-25 cells. - High expression in 5-8F cells, low expression in 6-10B cells	- Required for cell growth and proliferation. - Required for migration of nasopharyngeal carcinoma cells (5-8F and 6-10B). - Proliferation of FaDu hypopharyngeal squamous cells (FaDu) inhibited by midazolam that targets TRPM7.	[44,45,46]
Retinoblastoma	- Existence in 5-8F cells	- Required for cell proliferation. - Required for 5-8F cell migration.	[47]
Melanoma	- Expressed in cell lines	- Not reported.	[48,49]
Lung carcinoma	- Expressed in A549 cells	- Required for migration of A549 cells.	[50]
Erythroleukemia	- TRPM7-like currents in cell lines	- Not reported.	[51]
Colon cancer	- TRPM7 (Thr1482Ile) polymorphism	- TRPM7 (Thr1482Ile) polymorphism associated with elevated risk of both adenomatous and hyperplastic polyps. - Individuals with TRPM7 (Thr1482Ile) polymorphism with a high Ca:Mg ratio intake in diet at a relatively high risk of developing adenoma and hyperplastic polyps.	[52]
Leukemia	- Not reported	- Waixenicin inhibits T cell leukemia (Jurkat T lymphocytes) and rat basophilic leukemia cells (RBL1) through blocking TRPM7 channel activity.	[53]
Neuroblastoma	- Not reported	- In mouse neuroblastoma cells (N1E-115), TRPM7 promotes formation of Ca^2+^ sparking and invadosome by affecting actomyosin contractility independent from Ca^2+^ influx. - In vivo and in vitro studies using N1E-115 cells, TRPM7 promotes tumor metastasis in a mouse xenograft model and cell migration in Boyden chamber.	[54,55]
Ovarian carcinoma	- Somatic mutation S406C (Ser→Cys) in ovarian serous carcinoma	- Not reported.	[40]
Prostate cancer	- Expressed in human prostate cancer cell line DU145	- Increased Ca^2+^ to Mg^2+^ ratio in prostate cancer cells enhances TRPM7-mediated currents and promotes cellular entry of Ca^2+^, leading to increase in cell proliferation.	[56]
Glioblastoma	- Over-expressed in human glioblastoma cell line U87	- Carvacrol inhibits TRPM7 and suppresses glioblastoma cell proliferation, migration, and invasion - Xylokeletal B inhibits TRPM7 and suppresses glioblastoma cell proliferation and migration through PI3K/Akt and MEK/ERK signaling - Midazolam inhibits TRPM7-mediated current and suppresess TRPM7 expression, and induces cell cycle arrest and impairs proliferation	[57,58,59]

**Table 2:** Chemical modulators of TRPM7 channel activities as potential anti-cancer therapeutics.

Chemicals	Effects on TRPM7	Types of Cancer (Cell Lines)	Cellular Effects	References
Midazolam	Reduces expression of TRPM7, blocks TRPM7 channel activity (TRPM7 currents)	Human hypopharyngeal squamous cell carcinoma (FaDu), human glioma (MGR2)	Inhibits growth and proliferation with cell cycle arrest	[46,59]
Ginsenoside Rg3	Blocks TRPM7 channel activity (TRPM7 currents)	Human gastric adenocarcinoma (AGS)	Inhibits growth and survival (MTT-based viability assay: IC_50_ of 350 μM)	[42]
Ginsenoside Rd	Inhibits TRPM7 channel activity (TRPM7 currents)	Human breast adenocarcinoma (MCF-7), Human gastric adenocarcinoma (AGS)	Inhibits proliferation and survival (MTT-based viability assay: IC_50_ of 154 μM in MCF-7; IC_50_ of 131 μM in AGS)	[37]
Waixenicin A	Inhibits TRPM7 channel activity (TRPM7 currents: IC_50_ of 7 μM in 0 [Mg^2+^]_i_; 16 nM in 700 uM [Mg^2+^]_i_)	T cell leukemia (Jurkat), Rat basophilic leukemia (RBL1), Human gastric adenocarcinoma (AGS), Human breast adenocarcinoma (MCF-7), Mouse neuroblastoma (N1E-115)	Inhibits proliferation, inhibits invadosome formation	[38,53,54]
Carvacrol	Inhibits TRPM7 channel activity	Human glioblastoma (U87)	Inhibits survival, migration, and invasion (MTT-based viability assay: IC_50_ of 561 µM)	[57]
Xyloketal B	Inhibits TRPM7 channel activity (TRPM7 currents)	Human glioblastoma (U251)	Inhibits survival, proliferation and migration (MTT-based viability assay: IC_50_ of 287 µM)	[58]
NS8593	Inhibits TRPM7 channel activity (TRPM7 currents: IC_50_ 1.6 μM in [Mg^2+^]_i_, IC_50_ of 5.9 uM in 300 μM [Mg^2+^]_i_)	Human breast carcinoma (MDA-MB468)	Inhibits epidermal growth factor-induced vimentin expression (epithelial-mesenchymal transition)	[39,69]
